# Supplementary material for: Genetic Diversity and Population Structure of the Invasive Oriental Fruit Fly, Bactrocera dorsalis (Diptera: Tephritidae) in Burkina Faso
Source: Insects. 2024 Apr 23;15(5):298. doi: 10.3390/insects15050298 (PMC11122266; doi:10.3390/insects15050298)
Supplement: Supplementary file 1 [file insects-15-00298-s001.zip › insects-2916659-supplementary.pdf]

Supplementary Figures

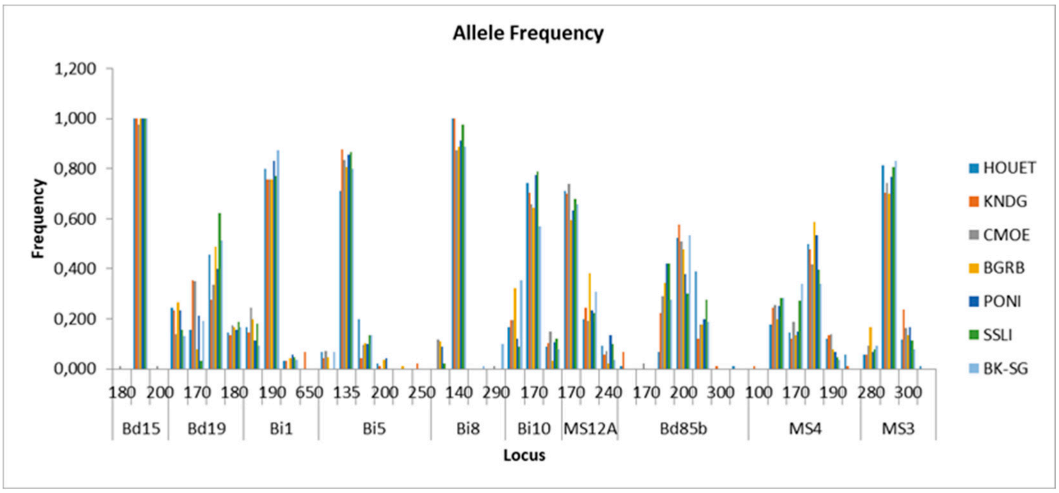

Figure S1: Allele frequencies by population with over loci

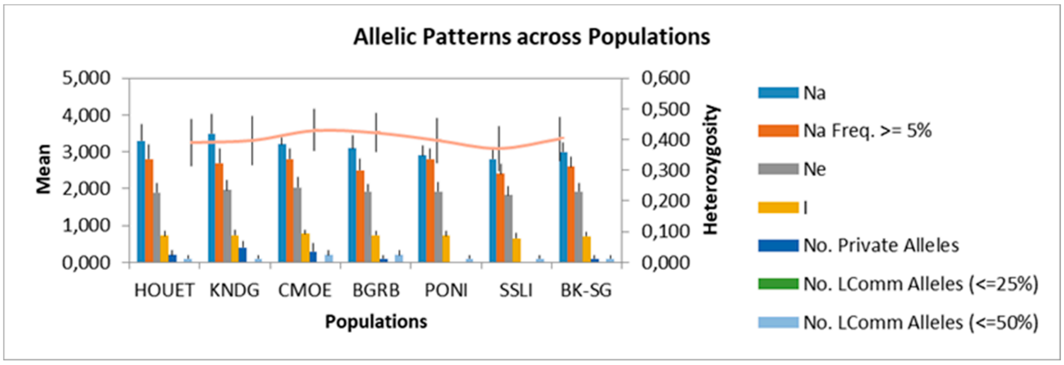

Figure S2: Allelic patterns across populations

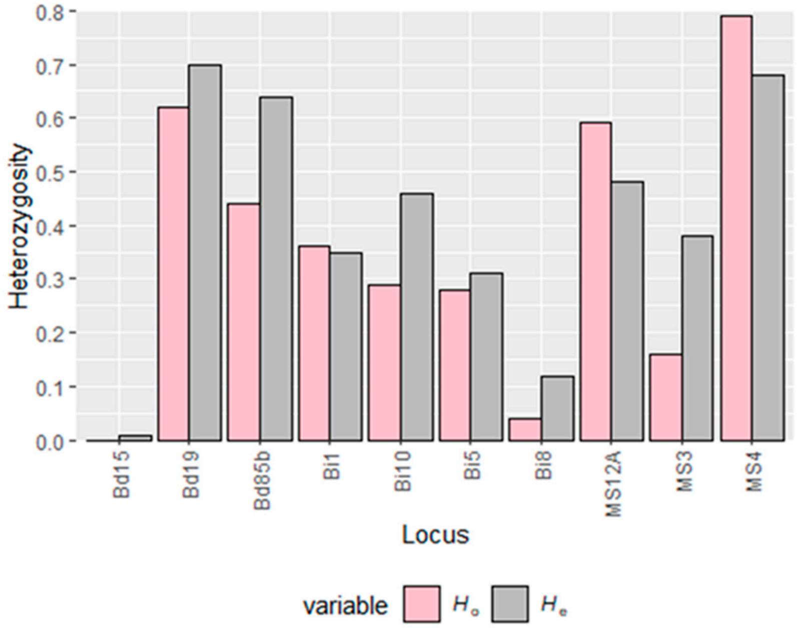

Figure S3: Comparison of observed and expected heterozygosity averages by locus

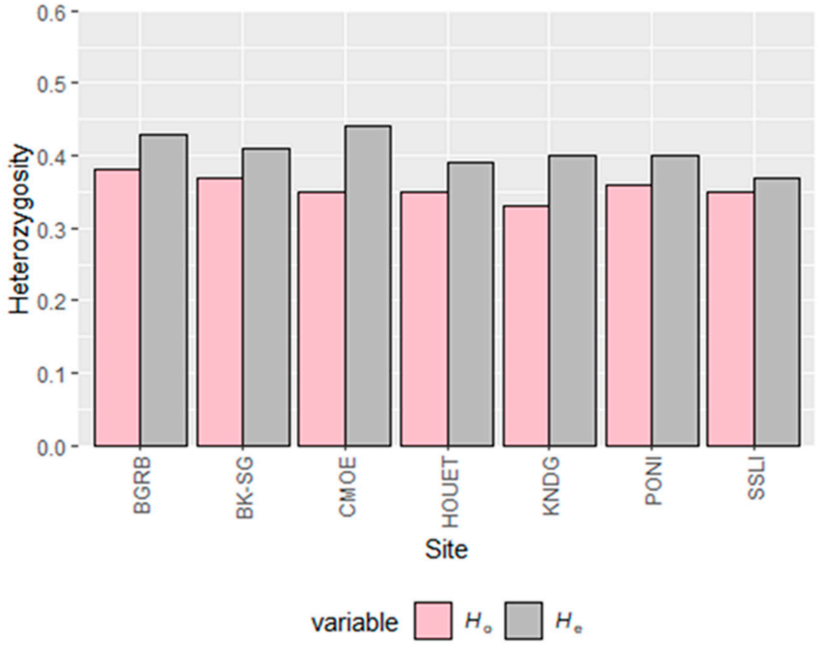

Figure S4: Comparison of observed and expected heterozygosity averages by sub-population

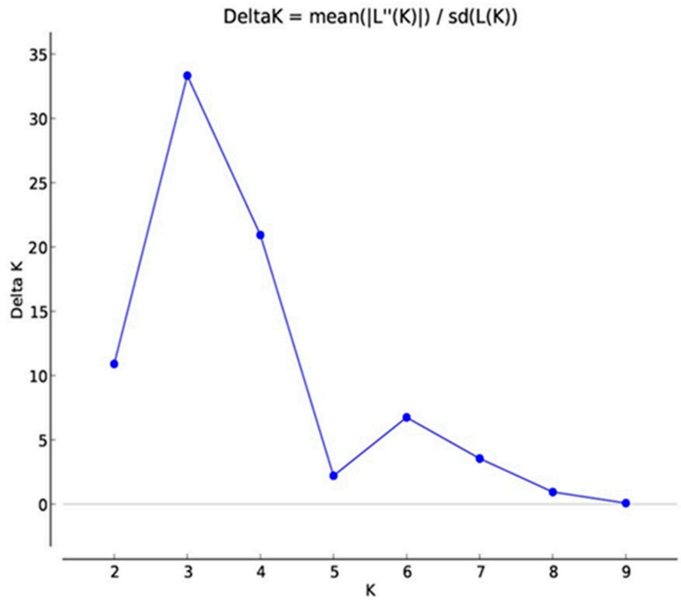

Figure S5: Evanno et al. (2005) plots for detecting the best number of K value [39].

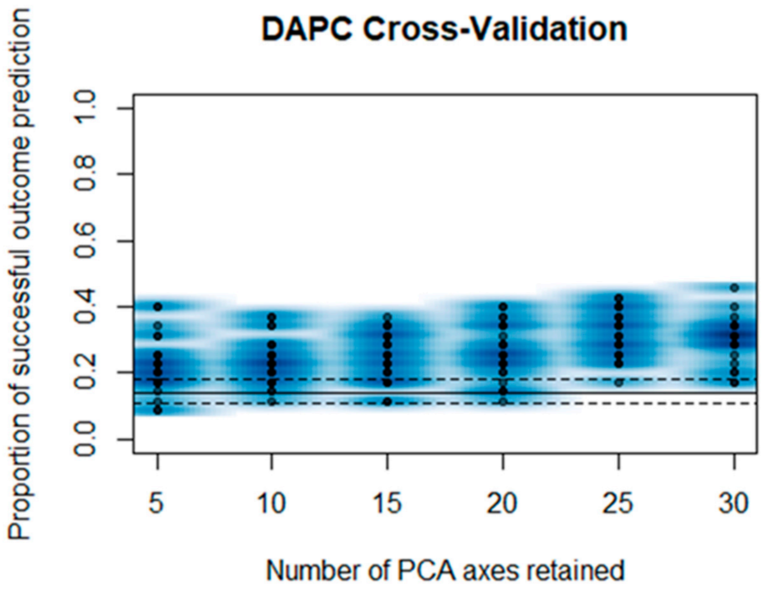

Figure S6: DAPC cross-validation plot for the best number of PCs

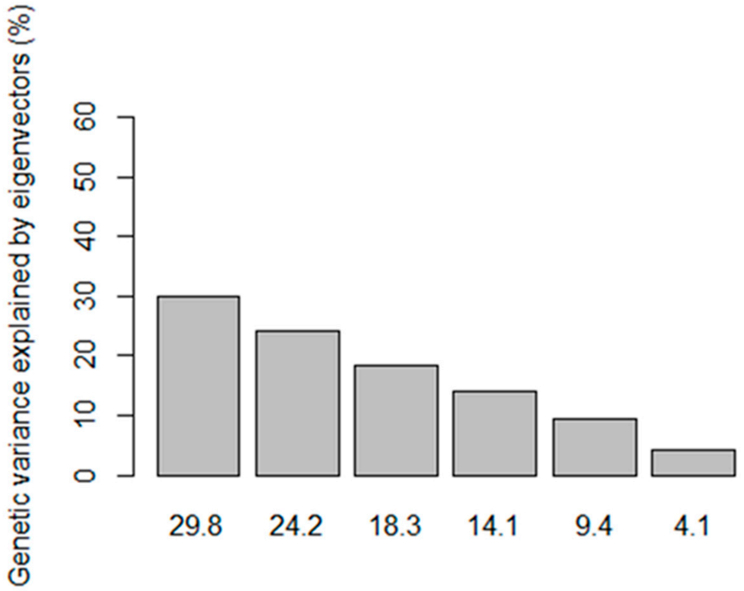

Figure S7: Genetic variance explained by eigenvectors

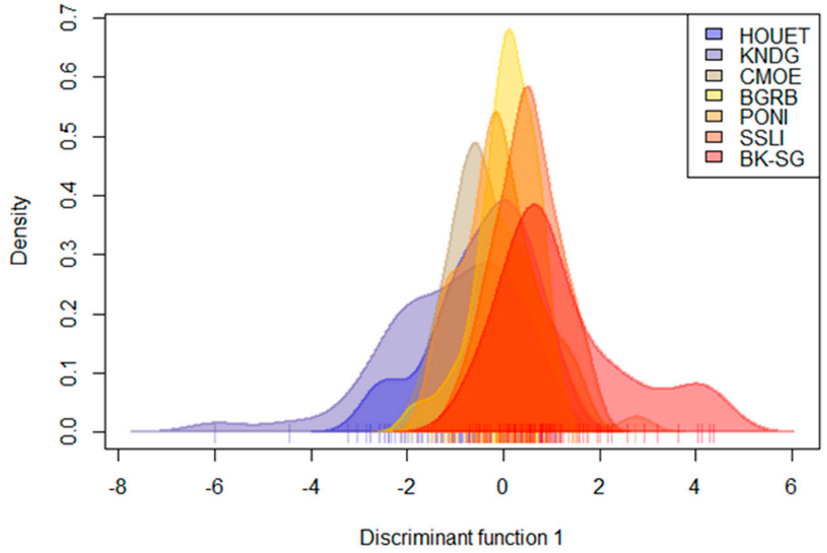

Figure S8: Individual density plot on the first discriminant function
